# The effect of family integrated care on the prognosis of premature infants

**DOI:** 10.1186/s12887-022-03733-0

**Published:** 2022-11-19

**Authors:** Hongyu Chen, Le Dong

**Affiliations:** 1 Department of Neonatology, Northwest Women and Children’s Hospital, Xi’an, China; 2 Department of Ophthalmology, Xi’an No.9 Hospital, No.151 East Section of South Second Ring Road, Beilin District, Xi’an, 710000 China

**Keywords:** FICare, Premature infant, Prognosis

## Abstract

**Background:**

The purpose of this study was to investigate the effect of family integrated care (FICare) on the prognosis of children hospitalized with preterm infants.

**Methods:**

Two hundred thirty preterm infants admitted to our hospital from January 2019 to April 2021 were enrolled in a prospective randomized study and divided into 115 cases in the intervention group and 115 cases in the control group according to the random number table method, and given the FICare and the conventional care, respectively. The duration of nasogastric tube retention, time to achieve total enteral nutrition, rate of weight gain, exclusive breastfeeding rate, length of hospital stay, growth and development, readmission rate, parental self-efficacy, family functioning and complications related to prematurity were compared between the two groups.

**Results:**

Compared with the control group, the intervention group had shorter nasogastric tube retention time, shorter time to achieve total enteral nutrition, higher exclusive breastfeeding rate, shorter time of hospital stay and better growth rate. Before the intervention, there was no difference in parental self-efficacy and family functioning between the two groups; after the intervention, the intervention group had higher parental self-efficacy and higher ratings of family functioning, and the difference was statistically significant. Compared with the control group, the intervention group had a lower readmission rate and significantly lower incidence of infection and choking.

**Conclusion:**

The FICare can shorten the time of nasogastric tube retention, shorten time to achieve total enteral nutrition and hospital stay, increase the rate of exclusive breastfeeding and the rate of weight gain, finally improve the prognosis of preterm infants and have a positive effect on parents.

## Introduction

According to the relevant data, the incidence of preterm birth in China is 4%-7%, and the trend is increasing year by year [1, 2]. Because preterm infants are less adaptable to the outside world and have weaker resistance, they are more susceptible to harmful factors. Therefore, preterm infants generally need to be cared for in hospital neonatal intensive care units (NICU) after birth [3]. In the NICU, infants are physically separated from their parents, which often has an impact on the physical, mental and emotional health of both parents and infants. Family-centered care is an approach to planning and delivering health services that encourages more parental involvement in the care of the infant. However, in the NICU, parents usually play only a supportive role and the majority of the infant's care is provided by NICU professionals [4]. Therefore, how to effectively establish a neonatal intensive care unit, maintain parent–child relationships for parents of preterm infants, develop parental caregiving skills, and increase breastfeeding rates are pressing issues for healthcare professionals to address. The Family Integrated Care (FICare) is a model that is an extension of the principles of family-centered care. It is a model through which parents are true partners in the care of their infants, even in the NICU. In FICare, the inclusion of parents on the care team goes far beyond just allowing parents to be present and observe their infant's care [5–7]. FIcare in single family rooms has been reported to decrease the incidence of late-onset sepsis in preterm infants and shorter length of their hospital stay [8]. A randomized controlled trial of infants in Alberta confirmed that FIcare is effective in reducing the length of hospital stay of preterm infants [9]. Yet, fewer domestic reports have been published on the application of the FICare in preterm infants in China. On this basis, we investigated the effect of FICare on the prognosis of children hospitalized with preterm infants and parental outcomes in this study.

## Materials and methods

### Clinical information

A prospective randomized study was conducted using 230 preterm infants admitted to our hospital from January 2019 to April 2021, divided into an intervention group and a control group of 115 cases each according to the random number table method. Intervention group: 55 females and 60 males; gestational age at birth ranged from 28 to 36 weeks, with a mean gestational age of (31.81 ± 2.23) weeks. Control group: 58 females and 57 males; gestational age at birth ranged from 29 to 36 weeks, with a mean gestational age of (32.06 ± 2.09) weeks. The general information of the two groups was comparable (*P* > 0.05), as shown in Table 1. The study protocol met the relevant requirements of the Declaration of Helsinki. All methods were performed in accordance with the relevant guidelines and regulations. Written informed consent has been obtained from the parents of subjects for this study and has got ethics approval and consent by the Research Ethics Committee of the Xi’an No.9 Hospital. The Ethical Approval Number was CKY2019-184.

**Table 1:** Comparison of general information

Items	Intervention group (*n* = 115)	Control group (*n* = 115)	X^2^/t	P
Gender (Male/Female, n)	55/60	58/57	0.157	0.692
Gestational age at birth (weeks)	31.81 ± 2.23	32.06 ± 2.09	-0.912	0.363
Born in this hospital (n)	100	95	0.842	0.359
Age at admission (h)	3.56 ± 0.23	3.68 ± 9.21	0.140	0.889
Admission weight (g)	1789 ± 377	1794 ± 386	-0.099	0.921
Admission head circumference (cm)	27.02 ± 1.23	27.04 ± 1.16	-0.127	0.899
Admission height (cm)	40.32 ± 2.19	40.38 ± 2.13	-0.211	0.833
Maternal illness during pregnancy (n)				
Diabetes	3	5	0.518	0.472
Hypertension	11	8	0.516	0.472
Infections	47	45	0.072	0.788
Thyroid disease	7	6	0.082	0.775
Disease diagnosis (n)				
Sepsis	21	19	0.121	0.728
Respiratory distress syndrome	72	70		
Perinatal asphyxia	26	28	0.097	0.756
Infection	31	33	0.087	0.769
Hypoglycemia	20	18	0.126	0.723
Pneumonia	68	66	0.072	0.789

Inclusion and exclusion criteria details are in Table 2.

**Table 2:** Inclusion and exclusion criteria

Inclusion criteria	Exclusion criteria
Gestational age between 28 and 36 weeks	Preterm infants require surgical intervention
Preterm infants without gastrointestinal diseases and eligible for breastfeeding	Premature infants need invasive ventilator therapy
All life signs were stable and remained stable for > 24 h	Premature infants with birth weight < 500 g
Parents are the main caregivers of premature infants, and those who can accompany them to the hospital on time	Those expected to be discharged within 1 week
Those whose guardians are able to communicate properly and who have signed an informed consent form	Mothers who have contraindications to breastfeeding or who have dropped out
	Premature infants with congenital diseases, hereditary diseases, cranio-cerebral injuries or vital organ insufficiency

### Methods

The control group was given conventional nursing interventions, which was to admit the preterm infant to the unaccompanied neonatal intensive care unit, and the respiratory management, nutritional management, and temperature management during the hospitalization were completed by professional staff, and all daily nursing care was implemented by nursing staff, and the daily afternoon 14:00–17:00 was the time of limited visitation, parents could visit through the glass window in the visiting corridor outside the neonatal intensive care unit ward or remote if the condition changes, the attending physician informed the parents of the child's disease condition and treatment plan, and the hospital corridor screen played a video of breastfeeding-related knowledge on a loop. At the time of discharge, the nursing staff provided regular verbal health education to parents of preterm infants or booklets on health education.

The intervention group was given FICare based on the care of the control group. The details include: 1. Establishing FICare implementation team and opening a separate ward dedicated to FICare for newborns. The implementation team includes 3 neonatologists (mainly responsible for clinical consultation and treatment of preterm infants, guidance on feeding methods and complementary food addition), 1 head nurse (responsible for staff deployment and materials supply), 2 nurse practitioners in charge (responsible for training and guidance of parents of preterm infants), 7 neonatal intensive care unit specialist nursing staff (mainly responsible for care of preterm infants, post-discharge follow-up and physical examination of preterm infants, health education on feeding and parenting knowledge), and 1 psychological counselor (mainly responsible for psychological consultation and psychological guidance of parents of preterm infants)0.2. Parent training. The FICare implementation team taught parents about knowledge related to preterm infants through lectures and PowerPoint slides in the NICU, while demonstrating daily care skills for preterm infants, hand hygiene, NICU escort system and neonatal feeding methods, etc. for about 45 min each time, for 3 consecutive times, and parents were able to repeat the key contents of the class and daily care skills operation is qualified. 3. After the child's condition stabilizes, the parents enter the family ward, and the medical and nursing staff again instruct the parents on life interventions, informing them of feeding patterns and skin care, etc. The parents stay with the child 24 h a day and are fully responsible for the child's daily life, and the medical and nursing team in the companion ward provides nursing knowledge, skills guidance and psychological support to the parents at all times. Only parents are allowed in this room, while others can observe the child by visiting.

### Observed indicators

The duration of nasogastric tube retention, duration of total enteral nutrition, rate of weight gain, rate of exclusive breastfeeding, length of hospital stay, growth and development, readmission rate, parental self-efficacy, family functioning and complications related to prematurity were compared between the two groups.

Rate of increase in body mass: [discharge body mass—admission body mass]/total length of stay.

Parental self-efficacy [10]: assessed using the Parenting Competence Scale (PSOC), which includes 2 subscales of satisfaction and parenting efficacy. In this study, only the Parenting Efficacy Scale was used to assess parental self-efficacy in assuming their role. Higher scores indicate higher levels of parental competence.

Family functioning [11]: The Family Functioning Assessment Scale (FAD) was used to assess family functioning, including 7 subscales of behavior control, emotional involvement, role, communication, problem solving, and total functioning. The total functioning subscale was used in this study to assess family functioning in general, and the lower the score, the better their family functioning.

Complications related to prematurity: hospital infections, choking, bloating, diarrhea, and gastric retention in both groups of preterm infants.

### Statistics

SPSS 21.0 software was applied to analyze the data. The measurement data such as mean ± SD were expressed by t-test; the count data were expressed as rate (%) by chi-square χ2 test. Differences were considered significant at *P* < 0.05.

## Results

### Comparison of nasogastric tube retention time, total enteral nutrition time, weight gain rate and hospitalization time between two groups of children

Compared with the control group, the intervention group had shorter nasogastric tube retention time, total enteral nutrition time, exclusive breastfeeding rate, hospitalization time, and faster weight gain, and the difference was significant (*P* < 0.05), as shown in Table 3.

**Table 3:** Comparison of the duration of nasogastric tube retention, duration of total enteral nutrition, rate of weight gain, rate of exclusive breastfeeding, and length of hospital stay between the two groups of children (mean ± sd)

Items	Intervention group (*n* = 115)	Control group (*n* = 115)	t	P
Duration of nasogastric tube retention (d)	12.03 ± 1.08	18.87 ± 1.13	-46.926	< 0.001
Duration of total enteral nutrition (d)	12.12 ± 2.03	18.76 ± 1.09	-30.904	< 0.001
Rate of weight gain (g/d)	22.87 ± 2.06	17.24 ± 2.12	20.424	< 0.001
Length of hospitalization (d)	18.89 ± 1.38	25.92 ± 1.41	-38.211	< 0.001
Exclusive breastfeeding rate (%)	104 (90.43%)	69 (60.00%)		< 0.001

### Comparison of growth and development of children in the two groups

Compared with the control group, the children in the intervention group had better growth and development in height, head circumference and weight, with significant differences (*P* < 0.05), as shown in Table 4.

**Table 4:** Comparison of growth and development between the two groups of children (mean ± sd)

Items	Time	Intervention group (*n* = 115)	Control group (*n* = 115)	t	P
Height (cm)	2 weeks of birth	43.41 ± 2.13	42.12 ± 2.09	4.636	< 0.001
	4 weeks of birth	46.87 ± 2.31	44.03 ± 2.03	9.904	< 0.001
Head circumference (cm)	2 weeks of birth	29.03 ± 1.19	28.14 ± 1.12	5.840	< 0.001
	4 weeks of birth	31.02 ± 1.13	29.67 ± 1.09	9.221	< 0.001
Body weight (g)	2 weeks of birth	1992 ± 387	1900 ± 295	2.027	0.044
	4 weeks of birth	2112 ± 382	2006 ± 376	2.121	0.035

### Comparison of parental self-efficacy between the two groups

**Fig. 1:**
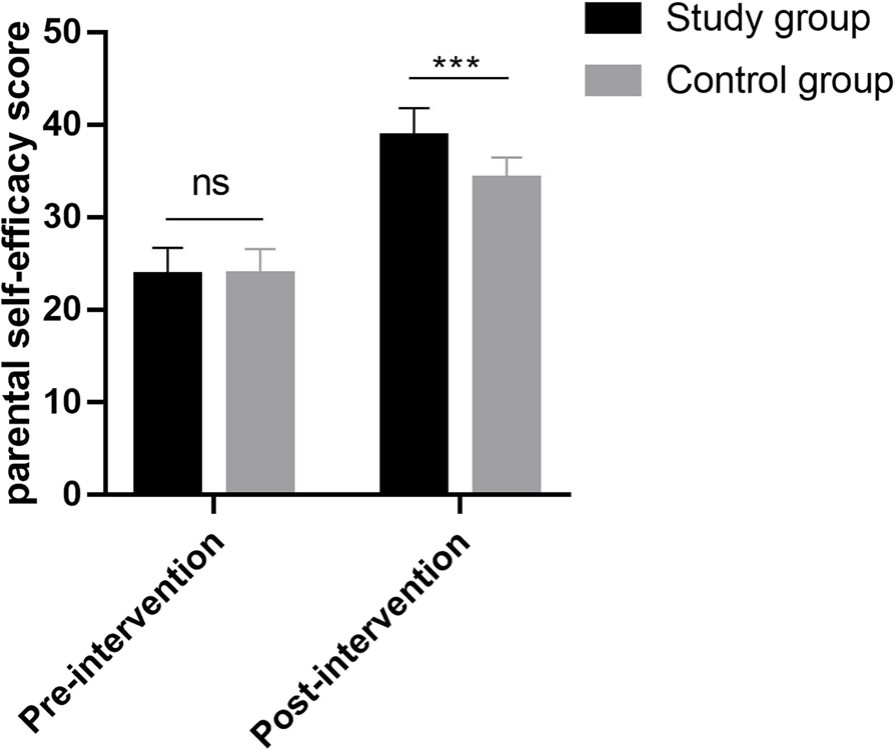
Comparison of parental self-efficacy between the two groups (mean ± sd)

Before the intervention, there was no statistically significant difference in parental self-efficacy between the two groups (*P* > 0.05); after the intervention, parental self-efficacy was higher in the intervention group compared with the control group, and the difference was statistically significant (*P* < 0.05), as shown in Fig. 1.

### Comparison of family function between the two groups

**Fig. 2:**
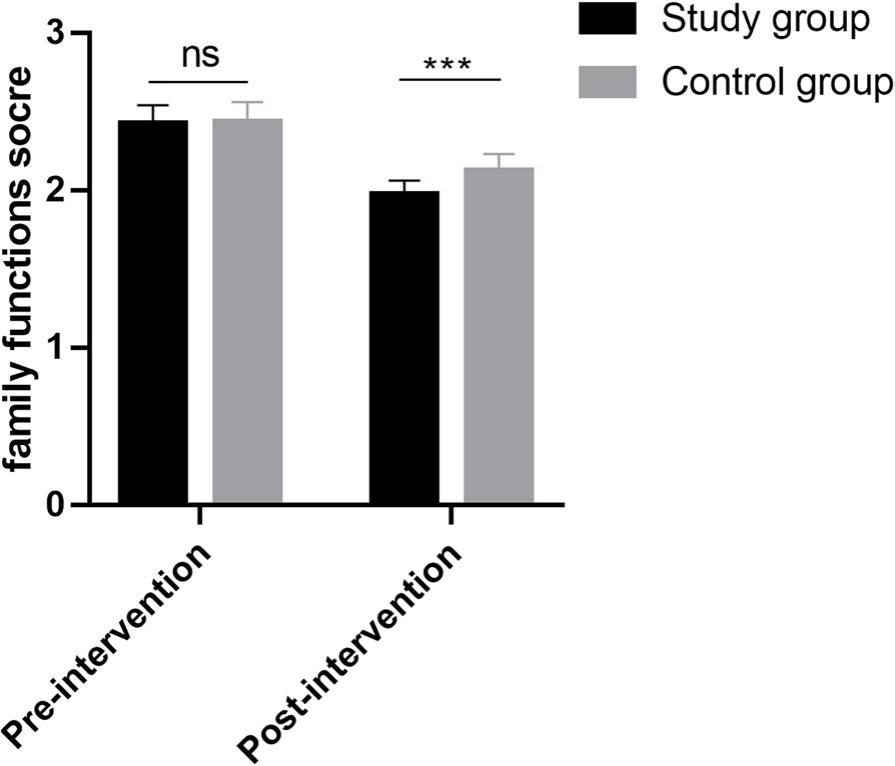
Comparison of family functions between the two groups (mean ± sd)

Before the intervention, there was no statistically significant difference in family function between the two groups (*P* > 0.05); after the intervention, family function was higher in the intervention group compared with the control group, and the difference was statistically significant (*P* < 0.05), as shown in Fig. 2.

### Comparison of readmission rates and complications related to prematurity between the two groups

Compared with the control group, there was a statistically significant difference in the readmission rate in the intervention group (*P* < 0.05); there was no statistically significant difference in the incidence of infection and choking in the two groups (*P* > 0.05), see Table 5.

**Table 5:** Comparison of readmission rates and complications related to prematurity between the two groups (%)

Items	Intervention group (*n* = 115)	Control group (*n* = 115)	t	P
Readmission rate n (%)	1 (0.01)	10 (0.09)	7.733	0.005
Infection	0	1 (0.01)	1.004	0.316
Choking	2 (0.02)	3 (0.03)	0.204	0.651

## Discussion

Premature infants often require lengthy hospital stays in NICU due to immature organ functioning that require them to establish respiratory stability and learn how to eat by mouth. During hospitalization they can also experience complications such as sepsis due to impaired immunological functioning [12, 13].The closed NICU can cause separation of mother and infant, resulting in difficulties for mothers to adapt to their new role, and even though nursing staff provides adequate health promotion education to parents, parents still lack the ability to care for their preterm infants, often leading to readmission of preterm infants due to inadequate care [14]. In many countries, parents of preterm infants are allowed to enter the NICU, kangaroo cuddling and family-centered care (FCC), all of which can improve the prognosis of patients and promote the development of family bonding, enabling parents to establish their parental role early and acquire knowledge and skills in neonatal care [15, 16]. At present, China has just started to implement family-involvement integrated management, which allows parents to visit in the NICU, but the visiting time is < 4 h, which hinders the establishment of emotional communication and intimate relationship between parents and newborns. Therefore, when exploring the implementation of family-involvement integrated management, we also attempted FICare in China.

In the NICU, as in the FCC, although the parents are involved in the care process, they usually only play a supporting role. FICare differs from FCC in that the parents are involved in all the processes of touching, feeding and bathing, and are the most important members of the care team in FIC, far beyond the observation of the medical staff present to care for the baby.

The results of this study showed that the intervention group had shorter nasogastric tube retention time, total enteral nutrition time, exclusive breastfeeding rate, shorter hospital stay, and faster weight gain compared with the control group (*P* < 0.05), suggesting that the FICare is beneficial to improve the prognosis level of the children. This may be due to the fact that preterm infants are transferred to the neonatal intensive care unit at birth, which separates mother and infant, and mothers worry about the prognosis of preterm infants, which is often accompanied by various negative emotions and is not conducive to their own recovery and that of the infant; at the same time, negative emotions can affect lactation, and with the lack of knowledge of mothers, preterm infants do not receive adequate breastfeeding, and all of these reasons are not conducive to the recovery of preterm infants and mothers themselves [17]. However, through FICare, close contact between mother and child, such as through touch, can relieve the mother's postnatal anxiety, promote lactation and uterine recovery, and the mother's caressing and cuddling of the newborn can increase the excitability of the vagus nerve in preterm babies, increasing the efficiency of food digestion and absorption, facilitating the absorption of nutrients and shortening the time to full intestinal feeding [18].

Height, weight, and head circumference are the most volatile of the neonatal growth indicators and the most readily available indicators of neonatal physical growth, and are the most important indicators of neonatal physical growth and nutritional status. Height, weight, and head circumference, especially weight, are directly related to the long-term prognosis of preterm infants and are important predictors of health outcomes in preterm infants. In a study by OK Kara et al., it was found that neonatal-centered "family-based" care was more beneficial to the growth and development of preterm infants [19]. The study by X Ding et al. also confirmed that integrated family care improved the feeding status of preterm infants in the NICU and contributed to the growth and development of preterm infants [20]. The results of this study showed that the growth and development of the intervention group were better compared with the control group (*P* < 0.05), suggesting that the FICare is beneficial to the growth and development of children hospitalized with preterm infants. This may be due to the fact that the FICare can provide time and space for close mother-infant contact and parental embrace feeding, which is more conducive to breastfeeding, and breastfeeding helps the absorption of nutrients, thus contributing to the growth and development of hospitalized newborns.

Self-efficacy is a judgment or belief about one's ability to successfully organize and complete a relevant task [21]. Our study showed that after the intervention, parental self-efficacy was higher in the intervention group compared to the control group, suggesting that the FICare can increase the self-efficacy of parents of children hospitalized with preterm infants. In this study, the implementation of FICare prompted parents to come to the ward 24 h actively to participate in the care of preterm infants, which increased parents' understanding of the disease and mastery of the condition. It can also compensate for the lack of parental role, effectively establish parent–child relationship, increase parent–child affection, meet the physical and psychological needs of parents and preterm infants, and reduce negative emotions. In addition, family functioning is the role played by family members in accomplishing various aspects of family tasks, and its ability to measure the effectiveness of family functioning is an important marker [22]. The strengths and weaknesses of family functioning are closely related to the physical and mental health of family members, and conversely, the health of family members can affect family functioning. Therefore, the results of this study showed that after the intervention, family functioning was higher in the intervention group compared with the control group (*P* < 0.05), suggesting that the FICare can improve the family functioning of parents of children hospitalized with preterm infants, which can improve the intimacy and cooperation among family members, create a relaxing and pleasant environment for parents, and promote their physical and mental health.

There are some limitations in our study. Since the development and application of the FICare requires support from various aspects such as human management components and hardware facilities, our data are relatively limited and need to be expanded into an in-depth study with a larger sample size. In addition, our data are mainly from preterm infants in China, and it is uncertain whether newborns in other countries around the world would also benefit from the FICare, which is a question that needs to be further explored later.

## Conclusions

In conclusion, the FICare can shorten the time to nasogastric tube placement, time to full enteral nutrition, exclusive breastfeeding rate, length of stay, increase weight gain rate, facilitate growth and development of preterm infants, improve parental self-efficacy and family functioning, reduce readmission rate, and not increase the incidence of infection and choking, and improve patient prognosis, which may be possible to promote the treatment of preterm children and bring benefits to parents.

## Data Availability

The datasets generated and analyzed during the current study are not publicly available but are available from the corresponding author on reasonable request.

## Abbreviations

FAD: Functioning Assessment Scale
NICU: Neonatal intensive care units
PSOC: Parenting Competence Scale
